# Differential effects of type of keyboard playing task and tempo on surface EMG amplitudes of forearm muscles

**DOI:** 10.3389/fpsyg.2015.01277

**Published:** 2015-09-01

**Authors:** Hyun Ju Chong, Soo Ji Kim, Ga Eul Yoo

**Affiliations:** ^1^Department of Music Therapy, Graduate School, Ewha Womans UniversitySeoul, South Korea; ^2^Music Therapy Education, Graduate School of Education, Ewha Womans UniversitySeoul, South Korea

**Keywords:** electromyography, keyboard-playing task, individuated finger movement, sequential finger movement, tempo

## Abstract

Despite increasing interest in keyboard playing as a strategy for repetitive finger exercises in fine motor skill development and hand rehabilitation, comparative analysis of task-specific finger movements relevant to keyboard playing has been less extensive. This study examined, whether there were differences in surface EMG activity levels of forearm muscles associated with different keyboard playing tasks. Results demonstrated higher muscle activity with sequential keyboard playing in a random pattern compared to individuated playing or sequential playing in a successive pattern. Also, the speed of finger movements was found as a factor that affect muscle activity levels, demonstrating that faster tempo elicited significantly greater muscle activity than self-paced tempo. The results inform our understanding of the type of finger movements involved in different types of keyboard playing at different tempi. This helps to consider the efficacy and fatigue level of keyboard playing tasks when being used as an intervention for amateur pianists or individuals with impaired fine motor skills.

## Introduction

Specific and intensive repetitions of finger movements effectively mediate the activation of corresponding muscle and brain areas, which leads to changes in functional muscular activities and cortical organization (Neistadt, 1994). Stroke patients showed increased test scores on manual function and improved speed and accuracy of motor movements after repetitive finger exercises via keyboard playing (Schneider et al., 2007, 2010; Rojo et al., 2011). Keyboard playing training led to significantly increased activation of motor areas in the brain and decreases in excessive contralateral or ipsilateral activation which was compromised to mediate the paretic hand prior to training (Rojo et al., 2011).

Keyboard playing as highly controlled finger movements involves multiple and complex motor movements (Zatsiorsky et al., 1998; Furuya and Altenmüller, 2013; Goebl and Palmer, 2013). Coordinated movements of different forearm muscles and kinematically fine control of involved joints operate to make optimal and efficient movements during keyboard playing (Zatsiorsky et al., 1998; Goebl and Palmer, 2013). Research suggests that, the type of keyboard playing task and degree and intensity of repetition is predictive of differential outcomes of fine motor development and rehabilitation (Westlake and Byl, 2013). However, the differential effects of specific target movements incorporated into keyboard playing have not yet been investigated. Although, previous studies have tended to apply individuated playing as a lower level task and sequential playing as a more complex task (Schneider et al., 2007, 2010), criteria for selection or modulation of the different levels of tasks have not been suggested.

Studies on kinematics and muscular activities of the fingers demonstrate that individuated or sequential finger motions involved in keyboard playing are related to different interactions between the fingers (Furuya et al., 2011). Research has repeatedly reported that fingers uninvolved in a specific keystroke are co-activated along with the finger intended to depress a key during individuated playing (Fish and Soechting, 1992; Häger-Ross and Schieber, 2000; Peleg et al., 2002; Furuya et al., 2011). Sequential playing for timely constrained movements elicits anticipatory movements for a single keystroke, and timing of sequences plays a critical role in control (Häger-Ross and Schieber, 2000; Furuya and Soechting, 2010; Bella and Palmer, 2011). Research has also demonstrated that, the type of sequential movement determines the motion trajectory and force of individual fingers (Bella and Palmer, 2011). Differences in maximum finger heights before keystrokes were found between tasks that required different fingers to be involved and that were performed at different speeds (Bella and Palmer, 2011).

As one of the indices for muscular performance, electromyography (EMG) studies present different patterns and amplitudes of muscular activation that are generated depending on the types of finger movements elicited by a multitude of sequences or combinations of keystrokes (Bella and Palmer, 2011; Furuya et al., 2011). Still, there are no conclusive guidelines for determining the level of finger movements for incorporation into keyboard-based education or intervention. Comparative analysis with regard to how relevant muscles are intensively involved in different target movements would present baseline data for developing keyboard playing strategies: expected muscular movements and intensity of the exercises involved in a specific task. Based on this, motor commands and the complexity level of the task could be taken into account. Therefore, this study examined whether there were differences in surface EMG amplitudes of finger flexor and extensor muscles for different keyboard playing tasks. The condition of keyboard playing was categorized into two primary task types: individuated and sequential finger movements. Sequential playing tasks are generated by a multitude of sequences or combinations of keystrokes and, accordingly elicit different finger movements. In this study, different types of sequential keyboard playing were examined: (a) involvement of adjacent fingers (i.e., from the thumb to the little finger) in a serial order and (b) involvement of non-adjacent fingers in a sequence. Also, the current experiment aimed to investigate whether muscular activation differed depending on the tempo of the keystrokes. The research questions were as follows:

1. Are there differences in mean sEMG activity levels depending on the keyboard playing task?
2. Are there differences in mean sEMG activity levels depending on the tempo of keyboard playing?

## Methods

### Participants

A total of 10 healthy male adults participated in this study. All participants were right-handed, and they reported no hand or finger injuries during the past 6 months. They also reported having less than 3 years of keyboard playing-related music education or training prior to the age of 10 and no professional music training within the past 10 years. Demographic information is summarized in Table 1. All participants gave written informed consent for participation in this study. The procedures and ethical issues were reviewed and approved by Institutional Review Board of Ewha Womans University (IRB No. 62-9).

**Table 1:** **Demographic information of participants**.

***N* = 10**	***M***	***SD***
Age (years)	34.0	5.8
BMI (kg/m^2^)	23.6	2.7
Keyboard-related experiences before age 10 (months)	6.1	10.1
Keyboard-related experiences within the past 10 years (months)	0.0	0.0

BMI: body mass index.

### Measurement

**Figure 1:**
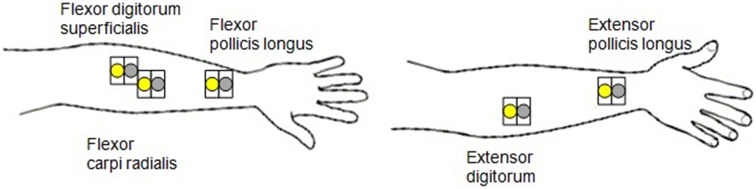
**Electrode placement**.

The interkeystroke interval in each playing task condition was measured via a MIDI-keyboard (YAMAHA DGX230) by computing all of the time intervals between two successive keystrokes in one trial of playing consisting of five keystrokes and averaging the intervals. The velocity of the finger movement during each keystroke was also measured. The surface electromyography (sEMG) data of forearm muscles were acquired via an eight-channel wireless QEMG-8 model (Laxtha Inc., South Korea). Five pairs of the Ag/AgCL surface electrodes (3M Inc., USA) were placed on three finger flexor muscles and two extensor muscles of each participant's dominant hand: flexor digitorum superficialis (FDS), flexor carpi radialis (FCR), flexor pollicis longus (FPL), extensor pollicis longus (EPL), and extensor digitorum (ED). The electrode placement is presented in Figure 1. The ground electrode was attached to the bony side of the back of the neck.

### Procedures

Each participant was instructed to maintain a posture with upright trunk position, forearm pronation parallel to the ground, and wrist neutral. Three tasks were presented to each participant: individuated playing, sequential playing in a successive pattern, and sequential playing in a random pattern (see Table 2). For individuated playing, participants were instructed to play five notes (C-D-E-F-G) using the thumb (T), index (I), middle (M), ring (R), and little (L) finger with a verbal cue indicating which finger to be played and the interval between verbal cues equal to approximately 1.5–2.0 s (*M* = 1.6, *SD* = 0.2). For two sequential playing tasks (i.e., the successive pattern and random pattern), the investigator explained the pattern to be played, and each participant practiced the pattern one to two times. For the successive pattern, each participant was instructed to depress five keys using T-I-M-R-L sequentially without pausing. Each participant was also instructed to sequentially play the random pattern of T-R-I-L-M with non-adjacent fingers. While the individuated playing task was presented at only a self-paced preferred tempo, both sequential playing tasks were tested at two different tempi; participants were asked to play each pattern at self-paced and fast tempo. In the self-paced tempo condition, each participant was instructed to play at a comfortable tempo. In the fast tempo condition, each participant was instructed to play the presented pattern as fast as possible. A total of five tasks were presented in a random order determined prior to the study. Each task was played three times, and each participant was allowed to pause for approximately 20–30 s between trials to prevent fatigue of their forearms.

**Table 2:** **Keyboard playing tasks**.

**Task**	**Pattern**	**Notes**	**Tempo**
Individuated playing	Depressing each key in isolation using T, I, M, R, and L	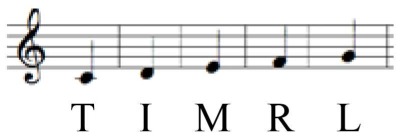	Self-Paced
Sequential playing	Depressing five keys successively without pausing using T, I, M, R, and L	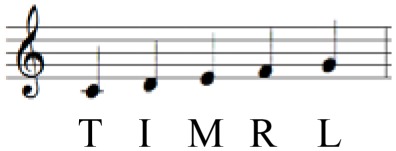	Self-Paced
			Fast
	Depressing five keys in a random pattern involving non-adjacent fingers in a sequence (T-R-I-L-M)	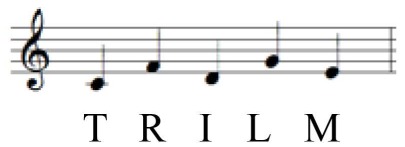	Self-Paced
			Fast

T, thumb; I, index finger; M, middle finger; R, ring finger; L, little finger.

### Data collection and analysis

Surface EMG values were pre-amplified with a gain of 244 and sampled at 1024 Hz. After the DC offset was removed, the signals were band-pass filtered at 20–450 Hz. The sEMG data were truncated at ±200 ms around each keystroke, and the root mean squared (RMS) values of each keystroke, without including the period of pause in the analysis, during each keyboard playing task at each tempo were calculated and averaged. Maximal voluntary contraction (MVC) for each muscle was obtained to normalize sEMG data by asking each participant to produce maximum force against a cylinder-like object grasped for 5 s. A one way repeated measures ANOVA was conducted to analyze the differences in the percentage MVC (%MVC) values between the playing tasks in each muscle, and a *post-hoc* comparison with Bonferroni correction was conducted to examine each paired comparison among the three playing tasks. For the effect of the tempo variable on sEMG values, a one way repeated measures ANOVA was also conducted.

## Results

### Keystroke-related data depending on the specified playing task pattern and tempo

For each of sequential playing tasks, interkeystroke intervals (IKI) and velocity were measured. When participants played the five keys using thumb-index-middle-ring-little fingers in a successive way, the mean IKI was 750 ms (*SD* = 200). For the random pattern that involved the finger movement of T-R-I-L-M, the mean IKI was 760 ms (*SD* = 130). When participants were instructed to play at fast tempo, the mean IKI was 200 ms (*SD* = 70) for the successive pattern and 210 ms (*SD* = 60) for the random pattern. These results indicate that participants performed the playing task at differentiated tempi as presented. A one way repeated measures ANOVA indicated that significant differences in the IKI were observed between the tempo conditions both during sequential playing in a successive way, *F*_(1, 9)_ = 16.030, *p* = 0.028, and sequential playing in a random way, *F*_(1, 9)_ = 69.632, *p* = 0.004.

With regard to the velocity of each keystroke, which is related to finger speed usually resulting in changes in loudness, the mean values measured during sequential playing at self-paced tempo were lower than those measured at fast tempo in both successive and random pattern conditions (see Table 3). However, a one way repeated measures ANOVA results showed that the differences did not reach statistical significance [*F*_(1, 9)_ = 1.944, *p* = 0.222 in the successive pattern playing, *F*_(1, 9)_ = 4.805, *p* = 0.080 in the random pattern playing].

**Table 3:** **Velocity of each keystroke during sequential playing**.

**Finger**	**Successive pattern**		**Random pattern**	
	**Self-paced *M (SD)***	**Fast *M (SD)***	**Self-paced *M (SD)***	**Fast *M (SD)***
Thumb	56 (10)	63 (14)	65 (10)	69 (15)
Index	58 (13)	73 (19)	67 (13)	75 (25)
Middle	56 (11)	71 (26)	65 (15)	84 (23)
Ring	52 (10)	69 (23)	61 (13)	76 (20)
Little	63 (8)	74 (29)	62 (11)	76 (20)
Mean	57 (10)	70 (22)	64 (12)	76 (20)

### Differences in EMG values depending on the specified playing task pattern

**Figure 2:**
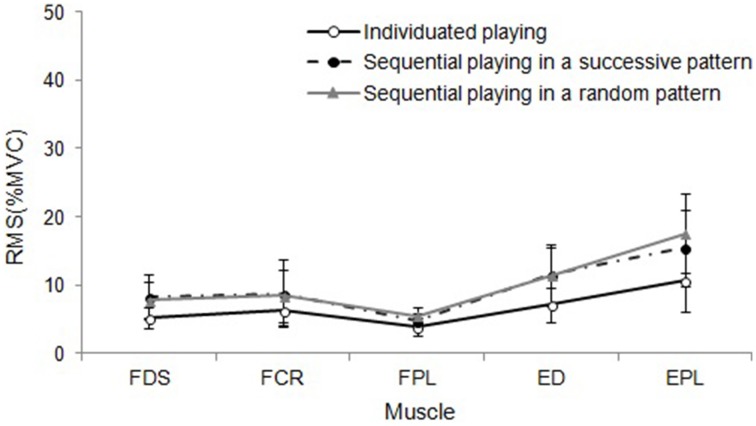
**Differential EMG activation of forearm muscles depending on playing task**. Error bar indicates the standard error of the mean.

When playing the keyboard at the self-paced tempo, muscle activation depending on the playing task was measured and compared with the use of averaged %MVC values for five keystrokes. The results of activation of each muscle depending on the playing task at self-paced tempo and the fingers pressed are displayed in Table 4 and Figure 2.

**Table 4:** **RMS EMG (%MVC) depending on finger and playing task in each muscle**.

	***M (SD)***					
	**T**	**I**	**M**	**R**	**L**	**Mean**
**FDS**						
In	4.3 (4.6)	5.8 (4.5)	5.4 (2.3)	6.8 (2.5)	4.0 (2.1)	5.2 (2.9)
S.S	5.7 (8.6)	7.4 (6.0)	8.8 (5.5)	12.4 (8.8)	7.5 (6.5)	8.3 (6.6)
S.R	5.9 (6.4)	8.2 (7.3)	9.5 (5.9)	10.0 (9.6)	5.4 (3.8)	7.8 (5.3)
**FCR**						
In	6.0 (4.7)	6.2 (4.7)	6.4 (4.8)	6.6 (5.5)	6.3 (4.6)	6.3 (4.5)
S.S	5.4 (2.9)	7.0 (6.5)	9.6 (11.5)	12.4 (17.6)	9.6 (13.7)	8.8 (10.0)
S.R	5.7 (2.7)	8.8 (8.6)	10.0 (10.0)	9.1 (9.7)	8.1 (8.2)	8.4 (7.7)
**FPL**						
In	4.4 (3.3)	3.8 (2.3)	4.2 (2.5)	3.9 (2.2)	3.9 (2.8)	4.0 (2.6)
S.S	4.9 (3.2)	4.3 (2.1)	5.0 (2.3)	5.1 (1.9)	4.6 (2.1)	4.8 (2.2)
S.R	5.8 (2.7)	5.7 (2.7)	5.5 (2.8)	5.1 (2.3)	5.4 (3.1)	5.5 (2.5)
**ED**						
In	8.0 (6.3)	6.7 (5.2)	7.8 (5.6)	6.2 (4.3)	7.1 (5.0)	7.1 (5.0)
S.S	11.3 (9.5)	10.7 (9.3)	14.1(12.1)	11.7 (9.5)	9.8 (7.4)	11.5 (9.1)
S.R	11.3 (8.0)	10.2 (6.5)	12.0 (9.8)	11.4 (8.7)	12.4 (9.8)	11.4 (8.3)
**EPL**						
In	11.3 (10.2)	9.7 (8.4)	11.9 (9.7)	9.5 (10.3)	11.3 (9.4)	10.7 (9.4)
S.S	15.3 (12.9)	13.8 (11.9)	17.5 (14.1)	13.0 (9.8)	18.1 (11.2)	15.5 (11.2)
S.R	15.6 (12.0)	17.4 (10.0)	22.6 (16.8)	13.8 (11.4)	18.7 (11.0)	17.6 (11.6)

FDS, flexor digitorum superficialis; FCR, flexor carpi radialis; FPL, flexor pollicis longus; ED, extensor digitorum; EPL, extensor pollicis longus; In, individuated playing; S.S, sequential playing in a successive pattern; S.R, sequential playing in a random pattern; T, thumb; I, index; M, middle; R, ring; L, little.

First, for the FDS, the primary finger flexor muscles, the results of a one way repeated measures ANOVA showed significant differences in the mean amplitudes (%MVC) depending on the playing task, *F*_(2, 8)_ = 5.737, *p* = 0.032. A *post-hoc* analysis with Bonferroni correction demonstrated that, the sequential playing task in random patterns produced significantly greater muscle activation than individuated playing (*p* = 0.044). Differences in EMG signals between individuated playing and successively sequential playing and between successively sequential playing and randomly sequential playing did not reach statistical significance. With regard to the level of activation in flexor carpi radialis, which is a primary muscle that mediates wrist flexion, there was no significant main effect of the playing task, *F*_(2, 8)_ = 0.969, *p* = 0.398, indicating little differences in EMG values for the different playing tasks. Thirdly, for the extrinsic flexor for the thumb, FPL, the main effect of the playing task was significant, *F*_(2, 8)_ = 7.985, *p* = 0.003, and a *post-hoc* analysis of paired comparison showed that the difference between individuated playing and sequential playing in a random pattern was significant (*p* = 0.010). The other paired comparison did not reach statistical significance. Also, the ED, the primary extrinsic extensor for the fingers, showed a significant main effect of the playing task, *F*_(2, 8)_ = 5.344, *p* = 0.015); a *post-hoc* comparison showed that although the difference between individuated and randomly sequential playing was the greatest, it did not reach statistical significance (*p* = 0.066). Lastly, there was a significant difference in the activation value of EPL depending on the playing task, *F*_(2, 8)_ = 10.335, *p* = 0.001. During a *post-hoc* analysis with Bonferroni correction, the difference between individuated playing and successively sequential playing (*p* = 0.020) and between individuated playing and sequential playing in a random pattern (*p* = 0.041) was found to be statistically significant.

In addition, the correlation between the velocity of each keystroke and sEMG value was measured during each playing task. Although, high positive correlation was observed in many of the pairs, the dimension of relationship in terms of statistical significance was yet inconsistent to generalize a conclusion (see Table 5). The results still imply that with larger samples, there would be a possible correlation between muscular activation of finger muscles and the amplitude and speed of actual finger movements.

**Table 5:** **Correlation between RMS EMG (%MVC) and the velocity of each keystroke**.

	**EMG (%MVC)**														
	**FDS**			**FCR**			**FPL**			**ED**			**EPL**		
	**In**	**S.S**	**S.R**	**In**	**S.S**	**S.R**	**In**	**S.S**	**S.R**	**In**	**S.S**	**S.R**	**In**	**S.S**	**S.R**
Vel.T	0.19	0.86^*^	0.24	0.37	0.36	0.20	0.85^*^	−0.11	0.14	−0.51	0.25	0.49	−0.43	0.60	0.10
Vel.I	0.32	0.93^**^	0.38	0.49	0.67	0.95^*^	0.76	0.02	0.03	−0.35	0.13	0.15	−0.49	0.68	−0.02
Vel.M	0.05	0.47	0.19	0.21	0.55	0.83^*^	0.94^**^	−0.27	0.79	−0.71	−0.02	0.34	−0.85^*^	0.68	0.14
Vel.R	0.27	0.72	−0.09	0.28	0.60	0.43	0.86^*^	−0.44	0.56	−0.42	0.03	0.22	0.14	0.55	0.37
Vel.L	0.14	0.87^*^	0.56	0.39	0.73	0.93^*^	0.79	−0.11	0.94^*^	−0.25	0.64	0.23	−0.15	0.75	0.38

Pearson's correlation was conducted. Vel, velocity of keystroke; S.S, sequential playing in a successive pattern; S.R, sequential playing in a random pattern.

* p < 0.05,

** p < 0.01.

### Differences in EMG values depending on the tempo

During sequential playing, sEMG data obtained from two different tempo conditions were compared. With regard to sequential playing in a successive pattern, playing at a faster tempo elicited significantly higher muscle activation than playing at self-paced tempo across four muscles of FDS [*F*_(1, 9)_ = 10.269, *p* = 0.009], FPL [*F*_(1, 9)_ = 4.149, *p* = 0.003], ED [*F*_(1, 9)_ = 12.904, *p* = 0.005], and EPL [*F*_(1, 9)_ = 10.113, *p* = 0.010]. The only exception was found in FCR; however, the degree of differences in sEMG data depending on the tempo observed in the FCR (*p* = 0.069) indicated possible differences in the measures with increased sample size.

**Figure 3:**
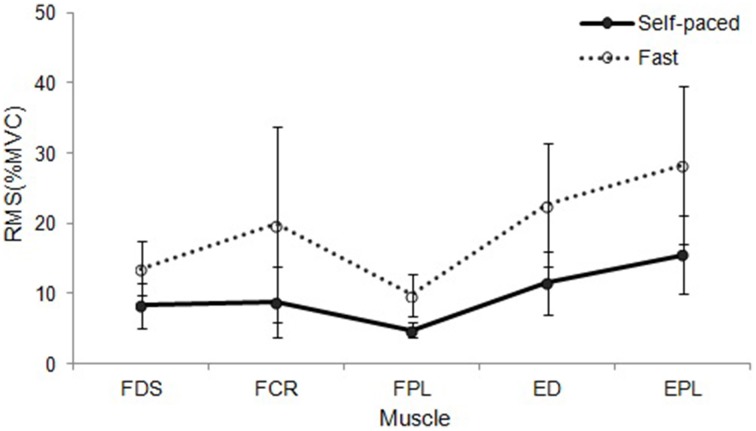
**RMS EMG (%MVC) depending on tempo during sequential playing in a successive pattern**. Error bar indicates the standard error of the mean.

**Figure 4:**
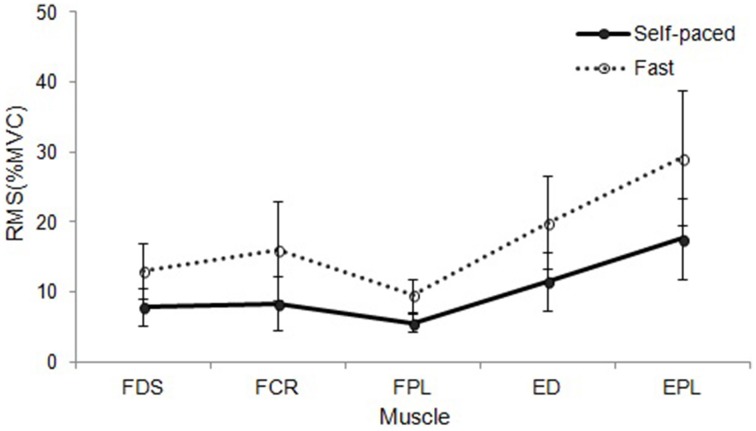
**RMS EMG (%MVC) depending on tempo during sequential playing in a random pattern**. Error bar indicates the standard error of the mean.

During random pattern playing, higher sEMG activation at faster tempo compared to self-paced tempo was observed in all muscles: FDS [*F*_(1, 9)_ = 9.598, *p* = 0.011], FCR [*F*_(1, 9)_ = 12.560, *p* = 0.005], FPL [*F*_(1, 9)_ = 29.891, *p* < 0.001], ED [*F*_(1, 9)_ = 19.557, *p* = 0.001], and EPL [*F*_(1, 9)_ = 16.692, *p* = 0.002]. The data are summarized in Table 6, and the different patterns of muscular activation depending on the tempo are presented in Figures 3, 4.

**Table 6:** **The effect of playing tempo during sequential playing on EMG activation in each muscle**.

	**Successive pattern** ***M*** **(*****SD*****)**		**Random pattern** ***M*** **(*****SD*****)**	
	**Self-paced**	**Fast**	**Self-paced**	**Fast**
FDS	8.34 (6.59)	13.55 (7.67)	7.79 (5.33)	12.98 (7.86)
FCR	8.81 (10.13)	19.85 (27.98)	8.36 (7.72)	15.89 (14.13)
FPL	4.77 (2.17)	9.79 (5.99)	5.51 (2.55)	9.51 (4.76)
ED	11.52 (9.08)	22.64 (17.69)	11.45 (8.29)	19.90 (13.35)
EPL	15.55 (11.24)	28.28 (22.64)	17.61 (11.59)	29.16 (19.18)

FDS, flexor digitorum superficialis; FCR, flexor carpi radialis; FPL, flexor pollicis longus; ED, extensor digitorum; EPL, extensor pollicis longus.

## Discussion

This study investigated the differential effects of playing pattern and keystroke tempo on the muscle activity of fingers. Significantly greater muscle activity was measured with sequential playing than with individuated playing. Increased motor commands were elicited in finger flexors and extensors for controlling timely sequenced finger movements. Increased co-activation across fingers occurred when each of the five fingers depressed a key sequentially, rather than when a specific keystroke with one finger was individually targeted. The literature supports that sequential playing involves anticipatory movements to determine and modulate appropriate timing and motion trajectories for the timely constraint implementation (Häger-Ross and Schieber, 2000; Furuya and Soechting, 2010; Bella and Palmer, 2011). The selection of task-specific keyboard playing for motor development or rehabilitation must consider that different levels of muscle activity are elicited from different keyboard-playing tasks.

While random pattern playing, which involves non-adjacent fingers in a sequence, produced significantly greater sEMG values than did the individuated playing, differences between successively sequential playing and individuated playing did not reach statistical significance. Sequential playing of non-adjacent movements may require more motor demands because of increased complexity in temporal and spatial constraint and, accordingly, the activity levels were found to significantly increase. Increased levels of sEMG were also found during playing sequentially in a successive pattern, which supports the research showing that a preceding finger affects the motion trajectory related to subsequent keystrokes and co-activation across fingers occur (Loehr and Palmer, 2009). However, a quite large degree of variability within the group limits the statistical significance for increased sEMG values in successive playing compared to individuated playing. In order to delineate the criteria for selecting keyboard playing tasks for individuals in need of fine motor skills training or hand rehabilitation, further studies should confirm the effects of the type and level of sequential playing task on muscular activity levels by applying a variety of combinations of non-adjacent fingers.

With regard to the muscles, the results showed that differences between individuated and sequential playing elicited similar patterns of sEMG activity level from related forearm flexors and extensors, except the flexor carpi radialis. Maintaining the position of the wrist is a prerequisite for performing a keyboard playing task, which explains why there were fewer changes in muscle activation for these muscles across the different playing tasks. In other words, insignificant differences in sEMG data from the flexor carpi radialis might be attributed to the fact that it is a primary muscle to mediate wrist flexion rather than finger flexion. In future studies, in order to corroborate the pattern of muscular activation and to address the limitations of surface EMG in terms of accurate distinction between closely-spaced small forearm muscles and according crosstalk between electrodes placed on adjacent muscle groups (Mogk and Keir, 2003), fine-wire EMG should be considered.

In addition to specified playing task patterns, tempo was also found to be a significant factor determining the level of muscle activation during keyboard playing. A faster tempo produced significantly greater sEMG values than did self-paced tempo during sequential playing tasks in the successive and random patterns. Research has demonstrated that pianists show increased finger peak height prior to the target keystroke (Goebl and Palmer, 2008; Bella and Palmer, 2011) and increased velocity of key contact (Goebl and Palmer, 2008; Furuya et al., 2012) at faster tempi. Increased tempo provides decreased anticipation time for subsequent keystrokes. Accordingly, greater finger movements at faster tempi can compensate for a temporal limitation to performing keyboard-playing tasks, by seeking enough tactile feedback and adjusting motor trajectories to ensure accuracy of targeted finger movements. Other studies have implied that faster tempo elicits increased co-activation across fingers, requiring greater motor command for independent control of finger movements (Häger-Ross and Schieber, 2000). Along with insignificant differences in the velocity of keystrokes between self-paced and fast tempo conditions, this study supported greater muscle activation being elicited with greater motor commands, not simply with greater amplitude or velocity of actual keystroke movements at faster tempi. Meanwhile, significantly high levels of positive correlations between some pairs of sEMG and MIDI-measured velocity values suggest that multidimensional analysis of keyboard-playing would be more informative in terms of the mechanism of a specific playing task. More systematic analysis with larger samples would address how functional load of muscles and actual movement are interrelated in further studies. Also, the limitation in the application of tempo variables was less control of tempi: participants performed the task at self-determined moderate and fast tempi according to the level of their fine motor skill. Although, the results showed that there were obvious differences between moderate and fast tempi, more controlled application of different tempi will indicate the threshold values for optimal changes in sEMG measures when varying the tempo of keystrokes.

## Conclusion

In summary, this study demonstrates that task-specific keyboard playing elicits different muscular activity. Higher sEMG activity levels were found with sequential keyboard playing than with individuated keyboard playing, and the movement sequences of non-adjacent fingers were observed to require significantly higher motor commands. In addition, intention, planning, and execution of playing at a faster tempo elicited greater muscular activation than those at a slower tempo. The findings of this study provide data on healthy adults without professional keyboard-related training for determining the type, level, and intensity of multi-finger movements when keyboard-playing is applied to finger movement exercises or keyboard instruction for beginner pianists or individuals with impaired fine motor skills by considering the expected muscular activation and fatigue.

Keyboard playing has been increasingly applied to not only healthy amateur pianists, but also individuals with decreased or impaired hand function, such as stroke patients or older adults (Schneider et al., 2007, 2010; Olafsdottir et al., 2008), in that repetitive and intensive finger movements have been proven effective in improving finger dexterity and fine motor control. In that the surface EMG recordings present biomechanical analysis of how motor commands brought forth affect task performance functionally, quantitative analysis of task-specific EMG signals in this study will help determine the expected muscular activation and the level of complexity of multi-finger patterns. Future studies, including brain imaging techniques, motion analysis or standardized hand function tests, will provide more conclusive data not only on how keyboard playing interventions should be designed, but also on how the interventions influence target populations over time.

## Author contributions

HC, SK, and GY contributed to study conception and design, data acquisition, analysis, and interpretation, and manuscript writing.

### Conflict of interest statement

The authors declare that the research was conducted in the absence of any commercial or financial relationships that could be construed as a potential conflict of interest.

## References

[B1] Bella S. D. Palmer C. (2011). Rate effects on timing, key velocity, and finger kinematics in piano performance. PLoS ONE 6:e20518. 10.1371/journal.pone.0020518 21731615 PMC3121738

[B2] Fish J. Soechting J. F. (1992). Synergistic finger movements in a skilled motor task. Exp. Brain Res. 91, 327–334. 1459234 10.1007/BF00231666

[B3] Furuya S. Altenmüller E. (2013). Flexibility of movement organization in piano performance. Front. Hum. Neurosci. 7:173 10.3389/fnhum.2013.00173 PMC3712142 23882199

[B4] Furuya S. Aoki T. Nakahara H. Kinoshita H. (2012). Individual differences in the biomechanical effect of loudness and tempo on upper-limb movements during repetitive piano keystrokes. Hum. Mov. Sci. 31, 26–39. 10.1016/j.humov.2011.01.002 21816497

[B5] Furuya S. Flanders M. Soechting J. F. (2011). Hand kinematics of piano playing. J. Neurophysiol. 106, 2849–2864. 10.1152/jn.00378.2011 21880938 PMC3234081

[B6] Furuya S. Soechting J. F. (2010). Role of auditory feedback in the control of successive keystrokes during piano playing. Exp. Brain Res. 204, 223–237. 10.1007/s00221-010-2307-2 20521031 PMC3179864

[B7] Goebl W. Palmer C. (2008). Tactile feedback and timing accuracy in piano performance. Exp. Brain Res. 186, 471–479. 10.1007/s00221-007-1252-1 18193412

[B8] Goebl W. Palmer C. (2013). Temporal control and hand movement efficiency in skilled music performance. PLoS ONE 8:e50901. 10.1371/journal.pone.0050901 23300946 PMC3536780

[B9] Häger-Ross C. Schieber M. H. (2000). Quantifying the independence of human finger movements: comparisons of digits, hands, and movement frequencies. J. Neurosci. 20, 8542–8550. Available online at: http://www.jneurosci.org/content/20/22/8542.full.pdf+html 11069962 10.1523/JNEUROSCI.20-22-08542.2000 PMC6773164

[B10] Loehr J. D. Palmer C. (2009). Sequential and biomechanical factors constrain timing and motion in tapping. J. Mot. Behav. 41, 128–136. 10.3200/JMBR.41.2.128-136 19201683

[B11] Mogk J. P. M. Keir P. J. (2003). Crosstalk in surface electromyography of the proximal forearm during gripping tasks. J. Electromyogr. Kines. 13, 63–71. 10.1016/S1050-6411(02)00071-8 12488088

[B12] Neistadt M. E. (1994). The effects of different treatment activities on functional fine motor coordination in adults with brain injury. Am. J. Occup. Ther. 48, 877–882. 10.5014/ajot.48.10.877 7825702

[B13] Olafsdottir H. B. Zatsiorsky V. M. Latash M. L. (2008). The effects of strength training on finger strength and hand dexterity in healthy elderly individuals. J. Appl. Physiol. 105, 1166–1178. 10.1152/japplphysiol.00054.2008 18687981 PMC2576040

[B14] Peleg D. Braiman E. Yom-Tov E. (2002). Classification of finger activation for use in a robotic prosthesis arm. Neural Syst. Rehabil. Eng. 10, 290–293. 10.1109/TNSRE.2002.806831 12611366

[B15] Rojo N. Amengual J. Juncadella M. Rubio F. Camara E. Marco-Pallares J.. (2011). Music-supported therapy induces plasticity in the sensorimotor cortex in chronic stroke: a single-case study using multimodal imaging (fMRI-TMS). Brain Inj. 25, 787–793. 10.3109/02699052.2011.576305 21561296

[B16] Schneider S. Münte T. Rodriguez-Fornells A. Sailer M. Altenmüller E. (2010). Music-supported training is more efficient than functional motor training for recovery of fine motor skills in stroke patients. Music Percept. 27, 271–280. 10.1525/mp.2010.27.4.271

[B17] Schneider S. Schönle P. W. Altenmüller E. Münte T. F. (2007). Using musical instruments to improve motor skill recovery following a stroke. J. Neurol. 254, 1339–1346. 10.1007/s00415-006-0523-2 17260171

[B18] Westlake K. P. Byl N. N. (2013). Neural plasticity and implications for hand rehabilitation after neurological insult. J. Hand Ther. 26, 87–93. 10.1016/j.jht.2012.12.009 23391829

[B19] Zatsiorsky V. M. Li Z. Latash M. L. (1998). Coordinated force production in multi-finger tasks: finger interaction and neural networking modeling. Biol. Cybern. 79, 139–150. 9791934 10.1007/s004220050466

